# Optimizing HIV-1 protease production in *Escherichia coli *as fusion protein

**DOI:** 10.1186/1475-2859-10-53

**Published:** 2011-06-30

**Authors:** Federica Volontè, Luciano Piubelli, Loredano Pollegioni

**Affiliations:** 1 Dipartimento di Biotecnologie e Scienze Molecolari, Università degli Studi dell'Insubria, via J.H. Dunant 3, Varese, 21100, Italy; 2 The Protein Factory, Centro Interuniversitario di Ricerca in Biotecnologie Proteiche, Politecnico di Milano and Università degli Studi dell'Insubria, via Mancinelli 7, Milano, 20131, Italy

## Abstract

**Background:**

Human immunodeficiency virus (HIV) is the etiological agent in AIDS and related diseases. The aspartyl protease encoded by the 5' portion of the *pol *gene is responsible for proteolytic processing of the *gag-pol *polyprotein precursor to yield the mature capsid protein and the reverse transcriptase and integrase enzymes. The HIV protease (HIV-1Pr) is considered an attractive target for designing inhibitors which could be used to tackle AIDS and therefore it is still the object of a number of investigations.

**Results:**

A recombinant human immunodeficiency virus type 1 protease (HIV-1Pr) was overexpressed in *Escherichia coli *cells as a fusion protein with bacterial periplasmic protein dithiol oxidase (DsbA) or glutathione S-transferase (GST), also containing a six-histidine tag sequence. Protein expression was optimized by designing a suitable HIV-1Pr cDNA (for *E. coli *expression and to avoid autoproteolysis) and by screening six different *E. coli *strains and five growth media. The best expression yields were achieved in *E. coli *BL21-Codon Plus(DE3)-RIL host and in TB or M9 medium to which 1% (w/v) glucose was added to minimize basal expression. Among the different parameters assayed, the presence of a buffer system (based on phosphate salts) and a growth temperature of 37°C after adding IPTG played the main role in enhancing protease expression (up to 10 mg of chimeric DsbA:HIV-1Pr/L fermentation broth). GST:HIVPr was in part (50%) produced as soluble protein while the overexpressed DsbA:HIV-1Pr chimeric protein largely accumulated in inclusion bodies as unprocessed fusion protein. A simple refolding procedure was developed on HiTrap Chelating column that yielded a refolded DsbA:HIV-1Pr with a > 80% recovery. Finally, enterokinase digestion of resolubilized DsbA:HIV-1Pr gave more than 2 mg of HIV-1Pr per liter of fermentation broth with a purity ≤ 80%, while PreScission protease cleavage of soluble GST:HIVPr yielded ~ 0.15 mg of pure HIV-1Pr per liter.

**Conclusions:**

By using this optimized expression and purification procedure fairly large amounts of good-quality HIV-1Pr recombinant enzyme can be produced at the lab-scale and thus used for further biochemical studies.

## Background

Retroviral proteins are synthesized as polyprotein precursors, which are subsequently processed by specific proteases. The aspartyl protease encoded by the human immunodeficiency virus (HIV) plays an essential role in viral maturation and is considered an attractive target for the treatment of acquired immunodeficiency syndrome (AIDS). The HIV-1 protease (HIV-1Pr) is encoded by the 5' portion of the *pol *gene and is responsible for processing the *gag *and *gag-pol *polyproteins to yield mature capsid proteins and the enzymes protease, reverse transcriptase, and integrase [1].

In the past, HIV-1Pr was synthesized chemically [2] or expressed by recombinant DNA technology in various heterologous systems [3]. In order to produce large amounts of HIV-1Pr, different strategies were investigated: i) it was produced by autocatalytic processing of a larger precursor [4,5]; ii) it was fused to a variety of proteins, e.g., β-lactamase [6], glutathione S-transferase (GST), maltose-binding protein [7], N-terminal portion of γ-interferon [8], or as His-tagged recombinant protein [9]; iii) codon usage, A+T richness at the 5' end of the coding region, and the promoter were optimized [10,11]; and iv) it was recovered by refolding of *E. coli *inclusion bodies [12,13]. Owing to the cytotoxicity (and low solubility) of this protease, it is difficult to obtain it in large quantities: in most cases the expression level was low and the recombinant HIV-1Pr could be detected only by immunoblotting (see Additional file 1, Table S1). After about 20 years of investigations, HIV-1Pr is still considered an important target for developing new drugs to cope with AIDS. Recently, for example, peptides displaying a sequence identical to those segments of the HIV-1Pr monomers associated with the local elementary structures, LES, have been proposed to destabilize the native structure of the protease. These new inhibitors may have a high genetic barrier for resistance as they bind HIV-1Pr through highly conserved residues which play an essential role in the protein folding process. The properties of promising inhibitors of HIV-1Pr monomer folding were investigated with the help of spectrophotometric assays and circular dichroism spectroscopy [14]. Unfortunately, these studies require milligram quantities of the viral protein in the soluble and active form and its commercial cost represents a main limit for academic studies.

The purpose of this study therefore was to construct a high and reproducible expression system for HIV-1Pr and to establish a convenient (simple and fast) purification procedure for obtaining fairly large amounts of the corresponding recombinant active protein at the lab-scale. In this paper we describe two new methods for obtaining HIV-1Pr that were established by producing different chimeric proteins, but also by optimizing both the expression in *E. coli *(mainly by acting on host strain selection and on medium composition) and the purification and maturation processes.

## Results and Discussion

### Expression of DsbA:HIV-1Pr

A synthetic cDNA encoding for a variant HIV-1Pr was designed to optimize codon usage for protein expression in *E. coli *and to avoid autoproteolysis and disulfide bridge formation (Additional file 1, Figure S1) [15]. Preliminary trials focused on the expression of untagged HIV-1Pr (by using pET24b(+) and pET26b(+) plasmids): in no case was HIV-1Pr expressed to a detectable level (see Additional file 1, Supplementary text).

The synthetic cDNA was then cloned in pET39b(+) plasmid. In this way, a chimeric protein could be produced that was constituted by the target protease fused with the full-length *E. coli *periplasmic dithiol-oxidase DsbA protein (Additional file 1, Figure S1A). Under standard conditions - *i.e*., BL21(DE3)pLysS cells induced at the early-exponential phase of growth and cell recovery after 1 hour of incubation at 37°C after adding IPTG - approx. 0.7 mg of DsbA:HIV-1Pr is produced per liter of fermentation broth.

**Figure 1:**
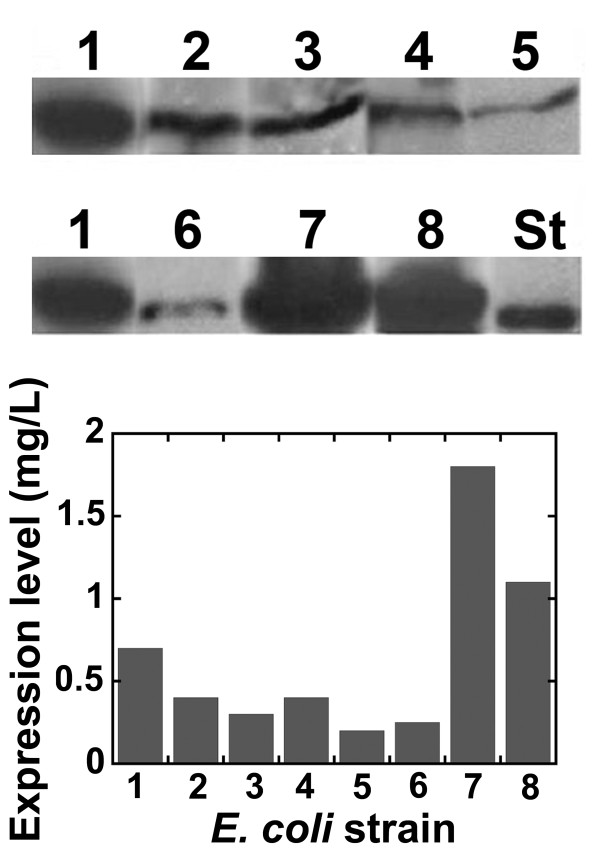
**Western blot analysis of DsbA:HIV-1Pr protein expression on total cell extracts of different *E. coli *strains containing the pET39-DsbA:HIV-1Pr plasmid.** As detected using anti-His-tag-specific antibodies. Cells were grown in LB medium at 37°C, protein expression was induced using 1 mM IPTG at early-exponential phase, and cells were harvested after 1 hour. *E. coli *strains are: 1: BL21(DE3)pLysS; 2: C41(DE3); 3: C43(DE3); 4: C41(DE3)pLysS; 5: C43(DE3)pLysS; 6: KRX; 7: BL21-CodonPlus(DE3)-RIL; and 8: BL21(DE3)Star. In all cases variability among replicate cultures was lower than 10%. The amount of cells corresponding to 1 mL of culture was loaded in each lane. St: 0.5 μg of His-tagged recombinant D-amino acid oxidase.

**Figure 2:**
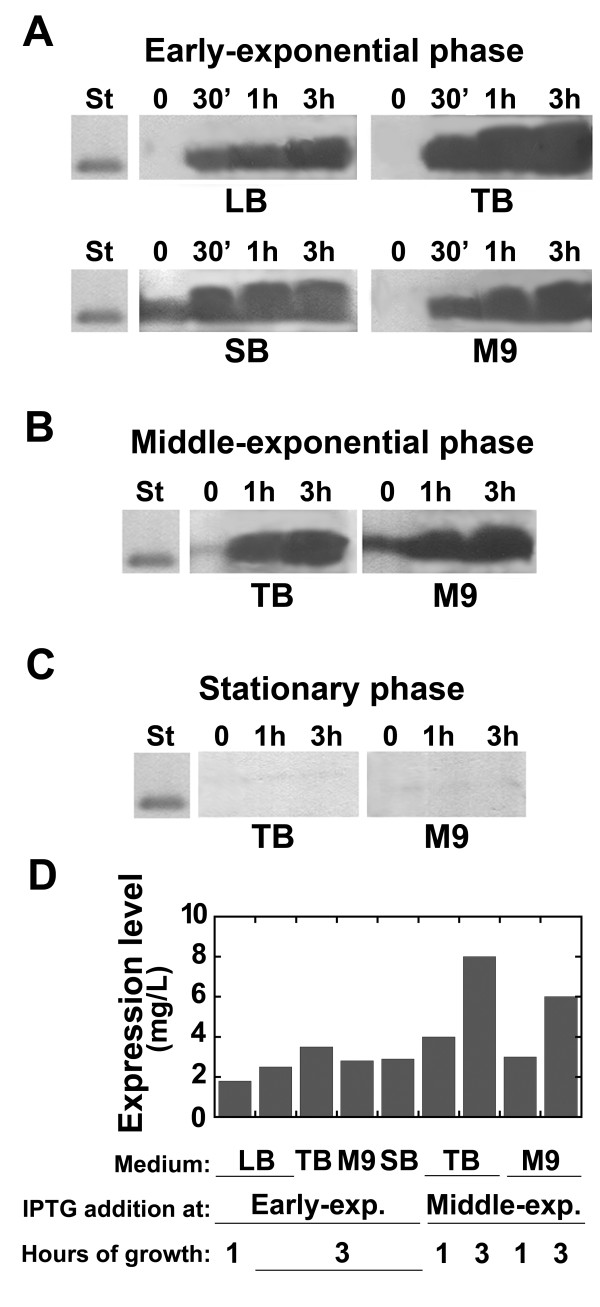
**Western blot analysis of DsbA:HIV-1Pr expression on total cell extracts of BL21-Codon Plus-(DE3)-RIL *E. coli *cells containing the pET39-DsbA:HIV-1Pr plasmid and using different cultivation media.** (Indicated below each panel): the cells were collected at different times after adding 1 mM IPTG (0, 0.5, 1, or 3 hours, as indicated above each lane). Protein expression induced at A) the early-exponential phase, B) the middle-exponential phase, and C) the stationary phase. An amount of cells corresponding to 1 mL (panel A) or 0.5 mL (panels B and C) of culture was loaded in each lane. D) Comparison of DsbA:HIV-1Pr production level obtained using different growth conditions. The first bar represents the best conditions identified using different *E. coli *strains (see Figure 1). In all cases variability among replicate cultures was lower than 10%. DsbA:HIV-1Pr expression was detected using anti-His-tag-specific antibodies. St: 0.5 μg of His-tagged recombinant D-amino acid oxidase.

**Figure 3:**
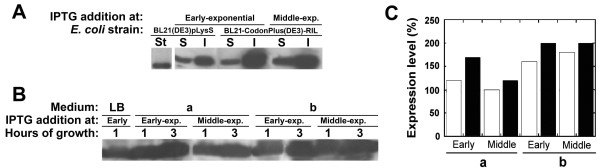
**Amount of soluble DsbA:HIV-1Pr and phosphate salts effect on chimeric protein production**. A) Comparison by means of Western blot analysis of the amount of DsbA:HIV-1Pr protein expression in the soluble (S) and insoluble (I) cell fractions as obtained after disruption of BL21(DE3)pLysS *E. coli *cells (growth in TB medium, protein expression induced at the early-exponential phase) or of BL21-Codon Plus-(DE3)-RIL *E. coli *cells (protein expression induced at the early and middle-exponential phase of growth). B) Western blot analysis of DsbA:HIV-1Pr protein expression in total cell extracts of recombinant BL21-Codon Plus-(DE3)-RIL *E. coli *cells grown in the presence of a phosphate salts buffer system. LB: cells grown in LB medium (control); a: cells grown in LB medium added of 6.78 g/L of NaH_2_PO_4 _and 3 g/L of KH_2_PO_4 _(such as in M9 broth); b: cells grown in LB medium to which 9.4 g/L of K_2_HPO_4 _and 2.2 g/L of KH_2_PO_4 _were added (such as in TB broth). Protein expression was induced in all samples with 1 mM IPTG; cells were harvested at 1 or 3 hours after adding IPTG. An amount of cells corresponding to 0.2 mL of culture was loaded in each lane. C) Summary results of DsbA:HIV-1Pr production (see panel B): the expression level of cells grown on LB medium is reported as 100% (= 1.8 mg/L). Cells were collected at 1 hour (empty bars) and 3 hours (black bars) from IPTG addition. Western blot analysis was carried out by using anti-His-tag-specific antibodies. St: 0.5 μg of His-tagged recombinant D-amino acid oxidase.

Expression trials were then carried out in LB medium containing 1% (w/v) glucose, growing the cells at 37°C and using different *E. coli *strains: BL21(DE3)pLysS, BL21-Codon Plus(DE3)-RIL, BL21-Star(DE3), C41(DE3), C41(DE3)pLysS, C43(DE3), C43(DE3)pLysS, and KRX (Figure 1) [16,17]. The BL21-Codon Plus(DE3)-RIL strain supplies extra copies of tRNA genes that are rare in *E. coli*; the BL21-Star(DE3) host strain allows good basal expression of the heterologous genes, mainly because of an increased stability of mRNA molecules, while C41, C43, and KRX *E. coli *strains are suitable for toxic heterologous protein expression because of a resistance to toxic proteins (C41 and C43 strains) or of a very stringent control of basal expression (KRX strain) [16,17]. At the same time, the effect of the time point of protein induction was also investigated by adding IPTG at the early-, middle-exponential, or stationary growth phase. Western blot analysis using anti-His-tag-specific antibodies on total cell extracts showed the highest DsbA:HIV-1Pr expression level for the BL21-Codon Plus(DE3)-RIL strain (Figure 1) and when IPTG was added at the middle-exponential phase of growth (see results in Figure 2B and 2D). Substantial expression of DsbA:HIV-1Pr chimeric protein was also observed by inducing protein synthesis at the early-exponential phase of growth, whereas no HIV-1Pr-associated signal was observed by adding IPTG at the stationary phase (Figure 2). In all cases, the recombinant fusion protein largely accumulated as inclusion bodies: a semi-quantitative analysis carried out by means of Western blot on the soluble fraction and the cell pellets estimated that ~ 80% of the expressed fusion protein is present in the insoluble fraction (see Figure 3A for cells grown in TB medium). On the basis of these results, subsequent investigations were carried out to optimize DsbA:HIV-1Pr expression using the BL21-Codon Plus(DE3)-RIL *E. coli *strain.

At first, the effect of cultivation medium composition both on the growth of recombinant BL21-Codon Plus(DE3)-RIL cells carrying the pET39-DsbA:HIV-1Pr plasmid and on the production of the chimeric protein was investigated. Cells grown in 5 different media (each of them added with 1% (w/v) glucose, see above) at 37°C were analyzed using the Gompertz equation (Additional file 1, Figure S2) [18]. The highest amount of cell paste (about 7.5 g of cells/L culture) was achieved in TB and SB media. Concerning HIV-1Pr production, protein expression was induced by adding 1 mM IPTG at cells grown on different media at the early- or middle-exponential phase (based on the growth curves as reported in Additional file 1, Figure S2); cell samples were withdrawn up to 3 hours after induction. Basal expression of recombinant chimeric HIV-1Pr (*i.e*., before adding IPTG) was observed only in SB medium. In all cases, the protein expression increased with time; the highest levels of DsbA:HIV-1Pr expression were obtained in TB and M9 medium and collecting the cells 3 hours after adding IPTG at the middle-exponential phase of growth (Figure 2).

The effect of medium composition on DsbA:HIV-1Pr production and the observation that a significant decrease in pH value was apparent during growth for all media, with the only exception of TB medium, suggest a positive role of the buffer system (based on phosphate salts) for increasing the expression yield. To test this hypothesis, cultures were grown in LB medium to which the same concentration of phosphate salts was added as present in TB or M9 media (~ 70 mM). Western blot analysis of total cell extracts showed a statistically significant increase in total DsbA:HIV-1Pr expression in the presence of phosphate salts, up to 2-fold vs. standard LB medium (Figure 3B, C).

**Figure 4:**
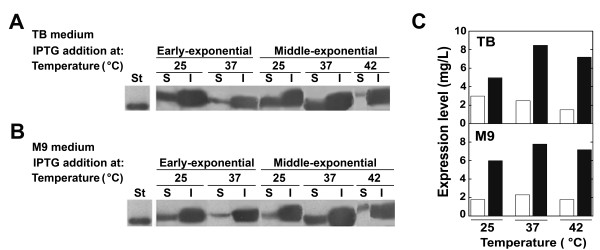
**Effect of temperature of growth after adding IPTG on the expression of DsbA:HIV-1Pr**. Western blot analysis of soluble (S) and insoluble (I) cell extracts as obtained after disruption of recombinant BL21-Codon Plus-(DE3)-RIL *E. coli *cells expressing DsbA:HIV-1Pr fusion protein grown in TB (A) or M9 (B) media at different temperatures. Protein expression was induced by adding 1 mM IPTG at the early or middle-exponential phase of growth. Experimental conditions as in Figure 2. C) Summary results of soluble (empty bars) vs. insoluble (black bars) level of DsbA:HIV-1Pr expression in cells induced at the middle-exponential phase of growth and using TB or M9 media (see panels A and B).

Finally, growth temperature can also affect the expression and the solubility of heterologous (recombinant) proteins expressed in *E. coli *[19]. Thus, the expression of DsbA:HIV-1Pr in cells grown in TB or M9 medium at 25, 37, and 42°C after adding IPTG was analyzed (Figure 4). The temperature did not affect the (in)solubility of the fusion protein: in all cases most of the expressed protein accumulated as inclusion bodies. Indeed, the highest level of total protein expression was observed growing at 37°C after adding IPTG at the middle-exponential growth phase.

The aggregated data show that the best conditions for lab-scale production of DsbA:HIV-1Pr are the use of *E. coli *BL21-Codon Plus(DE3)-RIL cells transformed with the pET39-DsbA:HIV-1Pr plasmid, grown at 37°C in TB or M9 medium, to which 1% (w/v) glucose has been added, induced during the middle-exponential phase, and collected after 3 hours (up to 10 mg of total chimeric protein/L).

### Purifying DsbA:HIV-1Pr fusion protein

**Figure 5:**
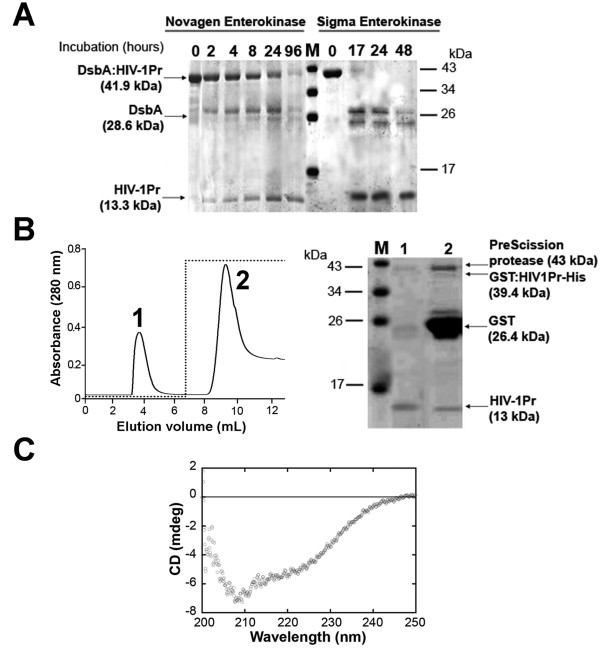
**Maturation of HIV-1Pr chimeric proteins by specific protease cleavage and isolation of HIV-1Pr**. A) SDS-PAGE Analysis of enterokinase maturation of DsbA:HIV-1Pr precursor. B) GSTrap on-column cleavage of GST:HIVPr-His by PreScission protease for 24 hours at 5°C. Left: elution profile. Peak 1 was eluted in PreScission buffer, after protease cleavage; peak 2 was eluted with 50 mM TrisHCl pH 8.0, 15 mM reduced L-glutathione. Right: SDS-PAGE analysis of eluted fractions. C) Far-UV CD spectrum of purified mature HIV-1Pr (0.1 mg/mL) in storage buffer.

The recombinant DsbA:HIV-1Pr was recovered from inclusion bodies using a specific solubilization and refolding procedure. Unsoluble material obtained from cell disruption was first washed twice with 2 M urea and 2% (v/v) Triton X-100, followed by a washing step with the same buffer without urea. Inclusion bodies were then solubilized using 6 M guanidium chloride under mild alkaline conditions (pH 8.0) in the presence of 2-mercaptoethanol as reducing agent and loaded on a HiTrap Chelating column previously equilibrated in the same buffer; the presence of 0.5 M NaCl and a low concentration of imidazole in the binding buffer reduces nonspecific interactions with the chromatographic matrix [20]. The refolded DsbA:HIV-1Pr was eluted, increasing the imidazole concentration up to 0.5 M. Lastly, a dialysis protocol comprising three different buffers was set up: at first the pH was decreased (from 8 to 7.5) and imidazole and EDTA concentrations were lowered, followed by a further decrease in pH (to 6.8) and imidazole, EDTA, and NLS concentrations, and finally equilibrating DsbA:HIV-1Pr in the storage buffer at pH 6.0, containing 5 mM EDTA, 0.01% Triton X-100, 10% (v/v) glycerol, and 1 mM 2-mercaptoethanol. In order to avoid precipitation/aggregation of the fusion protein, it is mandatory to change the pH gradually and to remove imidazole, EDTA, and NLS: dialysis of solubilized DsbA:HIV-1Pr against the storage buffer results in loss (precipitation) of a large part of the purified protein. By using this purification procedure the DsbA:HIV-1Pr fusion protein can be recovered with a purity grade higher than 95% (Figure 5A) and a > 80% recovery. Purification yield was about 8 mg and 6 mg of fusion protein/L culture for cells grown in TB or M9 media, respectively.

After dialysis, DsbA:HIV-1Pr fusion protein (~ 1 mg/mL) was digested with two different commercial enterokinases, as described in Methods section: in both cases full cleavage of DsbA:HIV-1Pr was achieved (Figure 5A). Despite the fact that different chromatographic methods (ion-exchange, PhenylSepharose, HiTrap chelating, etc.) were used, the DsbA and HIV-1Pr proteins were never fully separated. The best resolution was achieved by gel-permeation chromatography on Superdex 75, reaching a ≤ 80% of HIV-1Pr purity and an overall yield of ~2 mg HIV-1Pr per liter of fermentation broth. The activity of the purified HIV-1Pr was assayed on substrate III (Bachem), yielding a specific activity of 1.1 μmol/min mg protein. Indeed, the purified sample was fully stable at -20°C: no change in enzymatic activity was observed for up to 6 months.

### Expression of GST:HIVPr and HIV-1Pr purification

BL21-CodonPlus(DE3)-RIL *E. coli *cells transformed with pGST:HIV or pGST:HIV-His plasmids (see Additional file 1, Figure S1), grown in LB or TB broth (supplemented of 1% glucose) at 37°C, and to which 1 mM IPTG was added at the beginning of the exponential phase of growth (OD_600 nm _~ 1 or ~ 1.8 for LB or TB, respectively) produced approx. 0.5 mg of soluble fusion protein per liter of fermentation broth, corresponding to ~ 50% of the overall chimeric protein produced. Soluble GST:HIVPr and GST:HIVPr-His proteins were purified from the crude extract on a GSTrap column as described in the Methods section together with the free GST protein (see Additional file 1, Figure S3), suggesting an automaturation of the chimeric protein. On the contrary, when the His-tag was located at the N-terminal end of the fusion protein by using the plasmid pHis-GST:HIV, the His-GST:HIVPr fusion protein was fully accumulated as inclusion bodies up to ~ 5 mg of chimeric protein/L fermentation.

By performing the refolding of both GST:HIVPr-His and His-GST:HIVPr fusion proteins from inclusion bodies on the HiTrap affinity column (see Methods section), ~ 0.7 mg and 5 mg of protein per liter of fermentation could be isolated, respectively. The refolded GST:HIVPr-His sample contained substantial amounts of GST protein, exactly as happens for the soluble cellular fraction (Additional file 1, Figure S3). Indeed, this enzyme preparation was not cleaved by PreScission protease (both using the on- or the off-column procedure), thus preventing the obtainment of pure HIV-1Pr.

Concerning the isolation of HIV-1Pr from the chimeric proteins by PreScission protease treatment, the best result was obtained using the GST:HIVPr or GST:HIVPr-His soluble forms by on-column cleavage. The protein eluted in peak 1 of GSTrap column showed a high degree of purity (≥ 90% as judged by SDS-PAGE, Figure 5B), while the sample in peak 2 partly coeluted with the fusion partner GST because of the high HIV-1Pr hydrophobicity. An overall yield of ≥ 0.15 mg of pure HIV-1Pr/liter of fermentation broth was achieved (starting from 0.5 mg of the fused protein/liter). The activity of the purified HIV-1Pr was assayed on substrate III (Bachem), yielding a specific activity of 1.22 μmol/min mg protein. The Far-UV CD spectrum of the purified protease showed a ~ 30% β-sheet content (Figure 5C), as previously reported for folded HIV-1Pr [14].

## Conclusions

HIV-1Pr has been prepared by chemical synthesis [2] or as recombinant protein from *E. coli *cells, as reported by several groups [3-13]. In general the yield of purified, active HIV-1Pr from recombinant *E. coli *systems has been relatively low (≤ 1 mg protein/L broth, see Additional file 1, Table S1) and required complex purification procedures involving multiple chromatographic steps. Indeed, the procedures that resulted in higher expression yields (20-30 mg protein/L) suffered from low reproducibility [11-13]. In order to avoid cell death, overexpressed HIV-1Pr was also protein encapsulated with an engineered lumazine synthase capsid [21]. The difficulties in expressing recombinant HIV-1Pr are also made apparent by its high commercial cost (~ 25,000 €/mg protein). In the present paper, we report on a systematic investigation of HIV-1Pr expression in *E. coli *based on the combination of gene design, expression vector, and *E. coli *strain selection, and analysis of fermentation broth and conditions. The simple purification and processing procedure described in this work resulted in the production of ≥ 2 mg of HIV-1Pr/L with a ~ 80% purity following enterokinase digestion of resolubilized DsbA:HIV-1Pr and gel-permeation chromatography, or ~ 0.15 mg HIV-1Pr/L with a very high purity degree following PreScission protease cleavage of soluble GST:HIVPr and GSTrap chromatography purification.

In both cases, the purified HIV-1Pr retained its enzymatic activity and conformation (see Figure 5C for pure HIV-1Pr): these protease preparations represent a suitable tool to be employed at the academic level to investigate new strategies to tackle AIDS, i.e., for analyzing the potentialities of new classes of HIV-1Pr inhibitors [14]. This robust and reliable process for producing HIV-1Pr at lab-scale can eventually be scaled up to major volumes.

## Methods

### Design, synthesis, and cloning of cDNA coding for mature HIV protease

Synthetic cDNA coding for mature HIV-1Pr was designed by *in silico *back translation of the amino acid sequence reported in the database (GenBank Accession no. K03455). In order to eliminate autoproteolysis and to avoid disulfide bridge formation, four substitutions (Q7K, L33I, L63I and C95A) were inserted in the primary sequence (see Additional file 1, Figure S1) [15]. Synthetic cDNA was produced by Eurofins Medigenomix GmbH (Ebersberg, Germany), after optimizing the coding nucleotide sequence for expression in *E. coli*. The cDNA molecule was cloned into pET24b(+), pET26b(+), and pET39b(+) expression plasmids (Novagen, Madison, Wisconsin, USA) using *Nco*I (site introduced by PCR) and *Xho*I restriction sites. By cloning in pET39b(+) the target protein can be expressed as a fusion protein with the *E. coli *periplasmic protein dithiol oxidase (DsbA). An additional 6-residue C-terminal His-tag encoding sequence is also present in the resulting pET39-DsbA:HIV-1Pr plasmid.

The HIV-1Pr cDNA was also cloned in pGEX-6P-2 plasmid (GE Healthcare) using *BamHI *(site introduced by PCR) and *XhoI *restriction sites, giving the pGST:HIV expression vector. Two variants of this plasmid were further prepared: pGST:HIV-His, obtained using a specific PCR primer containing the 6-histidine codons before the *XhoI *cleavage site, and the pHis-GST:HIV vector prepared by mutagenesis using pGST:HIV as plasmid template and the primers HISGST_up (5'- CACACAGGAAACAGTATTCATGCATCACCATCACCATCACATGTCCCCTATACTAGG -3') and HISGST_dw (5'- CCTAGTATAGGGGACATGTGATGGTGATGGTGATGCATGAATACTGTTTCCTGTGTG -3').

### Strain, growth conditions, and enzyme expression

For protein expression, plasmids containing the HIV-1Pr cDNA were transferred into different *E. coli *strains: BL21(DE3)pLysS (Novagen, Madison, Wisconsin, USA), BL21-Codon Plus(DE3)-RIL (Stratagene, La Jolla, California, USA), BL21-Star(DE3) (Invitrogen, Carslbad, California, USA), KRX (Promega, Madison, Wisconsin, USA), and C41(DE3), C41(DE3)pLysS, C43(DE3), C43(DE3)pLysS (Lucigen, Middleton, Wisconsin, USA) host cells. Starter cultures were prepared using a single colony of *E. coli *cells carrying the recombinant plasmids in LB medium containing 1% (w/v) glucose and appropriate antibiotics: kanamicin (30 μg/mL final concentration) and chloramphenicol (34 μg/mL final concentration, only for BL21-Codon Plus(DE3)-RIL cells and for pLysS carrying strains) under vigorous shaking at 37°C. The following media were used [22,23]: Luria-Bertani (LB, 10 g/L bacto-tryptone, 5 g/L yeast extract, 5 g/L NaCl); Terrific Broth (TB, 12 g/L bacto-tryptone, 24 g/L yeast extract, 8 mL/L glycerol, 2.2 g/L KH_2_PO_4_, 9.4 g/L K_2_HPO_4_, corresponding to 70 mM total phosphate concentration); Super Broth (SB, 32 g/L bacto-tryptone, 20 g/L yeast extract, 5 g/L NaCl); M9 minimal medium (0.5 g/L NaCl, 1 g/L NH_4_Cl, 3 g/L KH_2_PO_4_, 6.78 g/L Na_2_HPO_4_, corresponding to 68 mM total phosphate concentration, 2 mM MgSO_4_, 0.1 mM CaCl_2_, 10 g/L glucose). Baffled (500 mL) Erlenmeyer flasks containing 100 mL of the different media were inoculated with the starter culture (initial OD_600 nm _= 0.05) and cells were grown at various temperatures with shaking (180 rpm). Protein expression was induced by adding 1 mM IPTG at different phases of the growth curve, as indicated in each case. Cells were harvested 30 min, 1 hour, or 3 hours after induction by centrifugation at 8000*g *for 10 min at 4°C. For protein purification trials, the cultures were prepared in 2 L Erlenmeyer flasks containing 700 mL of TB medium, inoculated with the starter culture (initial OD_600 nm _= 0.05), and cells grown at 37°C with shaking (180 rpm). Enzyme expression was induced by adding 1 mM IPTG at the middle-exponential phase and cells were harvested as described above 3 hours after adding the inducer. In all cases, cells were stored at -20°C.

For Dsb:HIV-1Pr purification, crude extracts were prepared by sonication (3-4 cycles of 30 s each, with a 30-s interval) on ice in 20 mM TrisHCl pH 8.0, 1 mM 2-mercaptoethanol, 1 mM phenylmethylsulfonylfluoride, and 10 μg/mL deoxyribonuclease I (3 mL/g cells). Samples were centrifuged at 34500*g *for 45 min at 4°C. As concerns the *E. coli *BL21-CodonPlus(DE3)-RIL cells transformed with pGST:HIV, pHis-GST:HIV, or pGST:HIV-His, the soluble fraction containing the fusion proteins was recovered by sonication on ice in PBS pH 7.4 (140 mM NaCl, 2.7 mM KCl, 10 mM Na_2_HPO_4, _1.8 mM KH_2_PO_4_), containing 1 mM phenylmethylsulfonylfluoride and 10 μg/mL deoxyribonuclease I (2.5 mL/g cells). After sonication, 1% (v/v) Triton-X 100 was added to the sample and it was incubated for 30 min at 4°C with mild shaking. Samples were centrifuged at 34500*g *for 45 min at 4°C and the soluble fraction (crude extract) was loaded on a GSTrap column.

### Solubilizing and purifying DsbA:HIV-1Pr to remove inclusion bodies

The insoluble fraction of *E. coli *cells expressing DsbA:HIV-1Pr after sonication (containing the inclusion bodies) was resuspended in 20 mM TrisHCl pH 8.0, 0.5 M NaCl, 2 M urea, and 2% (v/v) Triton X-100 (3 mL/g cells) and was sonicated (5 cycles of 10 s each, with 30-s intervals) and centrifuged (34500*g *for 45 min at 4°C); this procedure was repeated twice. The pellet was then resuspended in 20 mM TrisHCl, pH 8.0, 0.5 M NaCl, and 2% (v/v) Triton X-100 (3 mL/g cells) and centrifuged as above. The final pellet (inclusion bodies) was resuspended in 20 mM TrisHCl pH 8.0, 0.5 M NaCl, 5 mM imidazole, 6 M guanidium chloride, and 1 mM 2-mercaptoethanol (4 mL/g cells) and solubilized by gently shaking it for 1 hour at room temperature [20]. Samples were centrifuged at 34500*g *for 15 min at 4°C. The surnatant was filtered through a nonsterile Millex filter (pore size: 0.45 μm) (Millipore, Bedford, Massachussets, USA) and loaded on a HiTrap Chelating column (GE Healthcare, Uppsala, Sweden) containing Ni^2+ ^and equilibrated in 20 mM TrisHCl pH 8.0, 0.5 M NaCl, 5 mM imidazole, 6 M guanidium chloride, and 1 mM 2-mercaptoethanol. The column was then extensively washed with the same buffer and subsequently with 20 mM TrisHCl pH 8.0, 0.5 M NaCl, 20 mM imidazole, 6 M urea, 0.1% (w/v) N-lauroyl-sarcosine (NLS), and 1 mM 2-mercaptoethanol. The fusion protein was then refolded by slowly removing urea with a linear gradient (from 6 to 0 M) in 30-fold column volumes, and then eluted from the column using 20 mM TrisHCl pH 8.0, 0.5 M imidazole, 0.1% (w/v) NLS, 50 mM EDTA, and 5 mM 2-mercaptoethanol [20]. The eluted protein was dialyzed against the following buffers (1 hour each): 20 mM TrisHCl pH 7.5, 0.1 M imidazole, 0.1% (w/v) NLS, 20 mM EDTA, 5 mM 2-mercaptoethanol (dialysis buffer 1); 20 mM MES pH 6.8, 10 mM imidazole, 0.05% (w/v) NLS, 5 mM EDTA, 1 mM 2-mercaptoethanol (dialysis buffer 2); 20 mM MES pH 6.0, 5 mM EDTA, 0.01% (v/v) Triton X-100, 10% (v/v) glycerol, 1 mM 2-mercaptoethanol (storage buffer).

### Digestion of DsbA:HIV-1Pr with enterokinase

Two different recombinant enterokinases from bovine intestine were used: from Sigma-Aldrich (Saint Louis, Missouri, USA) and from Novagen (Madison, Wisconsin, USA) at 0.02 and 50 U/mg fusion protein, respectively. The digestion reaction was carried out for ~ 16 hours (overnight) at 25°C in storage buffer (see above) to which 20 μM CaCl_2 _was added.

### Purification of the GST:HIVPr fusion protein and PreScission protease cleavage

The crude extract containing the various GST:HIVPr fusion proteins was loaded on a GSTrap column (GE Healthcare, Uppsala, Sweden) equilibrated in PBS, pH 7.4. The column was then extensively washed with the same buffer and subsequently equilibrated in PreScission buffer (50 mM TrisHCl pH 7.5, 10 mM EDTA, 0.15 M NaCl, and 1 mM 2-mercaptoethanol) for the on-column cleavage procedure. PreScission protease was then loaded (70 μL of a 2 U/μL solution, GE Healthcare), the column was immediately closed and incubated for 24 hours at 5°C, followed by 2 hours at room temperature. The cleaved HIV-1Pr was then eluted in PreScission buffer.

After loading the crude extract, the GSTrap column was extensively washed in PBS pH 7.4 and then eluted with 50 mM TrisHCl pH 8.0 containing 15 mM reduced L-glutathione (Sigma Aldrich) for off-column PreScission protease cleavage (see above). The fusion protein was extensively dialyzed against PreScission buffer at 4°C, and then added of PreScission protease (20 U per mg of fusion protein). After incubation for 24 hours at 5°C followed by 2 hours at room temperature, the sample was loaded on a GSTrap column equilibrated in PreScission buffer, following the procedures described above. The eluted protein was dialyzed against the following buffers (1 hour each): 20 mM MES pH 6.8, 5 mM EDTA, 0.01% (v/v) Triton-X 100, 5% (v/v) glycerol, 1 mM 2-mercaptoethanol; 20 mM MES pH 6.0, 5 mM EDTA, 0.01% (v/v) Triton X-100, 10% (v/v) glycerol, 1 mM 2-mercaptoethanol (storage buffer).

### SDS-PAGE Electrophoresis and Western blot analysis

Proteins from total cell extract or from both soluble and insoluble cell fractions were separated by SDS-PAGE. For total cell extracts and for insoluble fractions after cell disruption, cell pellets were directly resuspended in an appropriate volume of Laemmli sample buffer. For Western blot analysis, proteins were transferred electrophoretically onto a nitrocellulose membrane and the fusion protein was detected using anti-His-tag mouse monoclonal antibodies (His-probe, Santa Cruz Biotechnology, Santa Cruz, CA, USA) and goat anti-mouse IgG HRP-conjugated antibodies (Santa Cruz Biotechnology, Santa Cruz, CA, USA). Recognition was then demonstrated by a chemiluminescence method (ECL Plus Western Blotting Detection System, GE Healthcare, Uppsala, Sweden). His-Tagged D-amino acid oxidase was used as positive control [24].

### HIV-1Pr Activity assay and CD measurements

A chromogenic substrate for HIV protease (substrate III, Bachem, Switzerland; with sequence H-His-Lys-Ala-Arg-Val-Leu-Phe(NO_2_)-Phe-Glu-Ala-Nle-Ser-NH_2_) was dissolved in 100% DMSO to reach a concentration of 10 mg/mL. The assay was performed in 20 mM sodium phosphate pH 6.0, 0.8 mM NaCl, 1 mM EDTA, and 1 mM dithiothreitol (freshly added). Each measurement was performed by recording the absorbance at 310 nm with a Jasco V-560 spectrophotometer, at 25°C, using cuvettes with a 3-mm optical path. The total volume sample was 70 μL; activity was assayed on a 100 μM substrate with a 10% DMSO final concentration. HIV-1Pr specific activity was calculated using the substrate extinction coefficient 500 M^-1 ^cm^-1^, as reported in [14].

Far-UV CD spectra were recorded in a Jasco J-815 spectrometer at 0.1 mg protein/mL and in storage buffer, as detailed in [25].

## List of abbreviations

DsbA: periplasmic *E. coli *dithiol oxidase; HIV-1Pr: aspartyl-protease encoded by HIV; IPTG: isopropyl-β-D-thiogalactopyranoside; NLS: N-lauroyl-sarcosine; GST: glutathione S-transferase.

## Competing interests

The authors declare that they have no competing interests.

## Authors' contributions

LP conceived the project and wrote the paper. FV produced the expression plasmids, performed the overexpression trials, purified HIV-1Pr from DsbA:HIV-1Pr and GST:HIVPr precursors, analyzed the data and helped write the paper. LuPi performed expression experiments on the DsbA:HIV-1Pr, purified HIV-1Pr from the fusion partner, and helped write the paper. All authors have read and approved the final manuscript.

## Supplementary Material

Additional File 1 **Supplementary HIV-1Pr.PDF**. Supplementary text describes the expression trials using pET24b(+) or pET26b(+) plasmid. Figure S1 describes the amino acid sequence of the chimeric proteins and the mature HIV-1Pr used in this work. Figure S2 reports the growth curve of BL21-Codon Plus-(DE3)-RIL *E. coli *cells carrying the pET39-DsbA:HIV-1Pr plasmid in different media. Figure S3 reports the SDS-PAGE analysis of the purification of GST:HIV-1Pr protease fusion forms. Table S1 summarizes published results concerning the production of recombinant HIV-1Pr in different heterologous hosts. Click here for file
