# Supplementary material for: Optimizing HIV-1 protease production in Escherichia coli as fusion protein
Source: Microb Cell Fact. 2011 Jun 30;10:53. doi: 10.1186/1475-2859-10-53 (PMC3141379; doi:10.1186/1475-2859-10-53)
Supplement: Additional File 1 — Supplementary HIV-1Pr.PDF. Supplementary text describes the expression trials using pET24b(+) or pET26b(+) plasmid. Figure S1 describes the amino acid sequence of the chimeric proteins and the mature HIV-1Pr used in this work. Figure S2 reports the growth curve of BL21-Codon Plus-(DE3)-RIL E. coli cells carrying the pET39-DsbA:HIV-1Pr plasmid in different media. Figure S3 reports the SDS-PAGE analysis of the purification of GST:HIV-1Pr protease fusion forms. Table S1 summarizes published results concerning the production of recombinant HIV-1Pr in different heterologous hosts. [file 1475-2859-10-53-S1.PDF]

Supplementary text

Expression of untagged HIV-1Pr

When growing cultures of recombinant BL21(DE3)pLysS or KRX *E. coli* cells carrying the HIV-1Pr gene cloned in pET24b(+) (a useful plasmid for heterologous protein expression in the bacterial cytoplasm) [22, 23] on LB medium at 37 °C, cell lysis was observed even before protein expression was induced by IPTG. Addition of 1% (w/v) glucose to the cultivation medium, a strategy commonly used to minimize basal expression (*i.e.*, before induction) [19], effectively overcame cell lysis but also prevented the expression of recombinant HIV-1Pr (data not shown).

HIV-1Pr cDNA was also cloned into the pET26b(+) vector, by which the heterologous protein can be translocated in *E. coli* periplasm because of the fusion with the pelB sequence (which is then removed by a specific peptidase). This plasmid was used to express the C-terminal His-tagged HIV-1Pr in BL21CodonPlus(DE3)-RIL *E. coli* cells grown in LB or TB broth (supplemented with 1% glucose) at 37 °C, and to which 1 mM IPTG was added at the beginning of the exponential phase of growth (OD_{600nm} ~ 1 or ~ 1.8 for LB or TB, respectively). Growth temperature after induction and time of incubation after adding IPTG were also varied: in no case was HIV-1Pr expression achieved (as judged by Western blot analysis, data not shown).

A

1 MKKIWLALAG LVLAFSASAA QYEDGKQYTT LEKPVAGAPQ VLEFFSFFCP HCYQFEEVLH 60

61 ISDNVKKKLP EGVKMTKYHV NFMGGDLGKD LTQAWAVAMA LGVEDKVTVP LFEGVQKTQT 120

121 IRSASDIRDV FINAGIKGEE YDAAWNSFVV KSLVAQQEKA AADVQLRGVP AMFVNGKYQL 180

181 NPQGMDTSNM DVFVQQYADT VKYLSEKKGS TSGSG**HHHHH H**SAGLVPRGS TAIGMKETAA 240

241 AKFERQHMDS PDLGT**DDDDK** SPGFSSTMVM PQVTLWKRPL VTIKIGGQLK EALLDTGADD 300

301 TVIEEMSLPG RWKPKMIGGI GGFIKVRQYD QIIIEICGHK AIGTVLVGPT PVNIIGRNLL 360

361 TQIGATLNFLE**HHHHHH**

B

1 MSPILGYWKI KGLVQPTRLL LEYLEEKYEE HLYERDEGDK WRNKKFELGL EFPNLPYYID 60

61 GDVKLTQSMA IIRYIADKHN MLGGCPKERA EISMLEGAVL DIRYGVSRIA YSKDFETLKV 120

121 DFLSKLPEML KMFEDRLCHK TYLNGDHVTH PDFMLYDALD VVLYMDPMCL DAFPKLVCFK 180

181 KRIEAIPQID KYLKSSKYIA WPLQGWQATF GGGDHPPKSD **LEVLFQGP**LG SMPQVTLWKR 240

241 PLVTIKIGGQ LKEALLDTGA DDTVIEEMSL PGRWKPKMIG GIGGFIKVRQ YDQIIIEICG 300

301 HKAIGTVLVG PTPVNIIGRN LLTQIGATLN F**HHHHHH**

Figure S1 Amino acid sequence of the chimeric proteins and of the mature HIV-1Pr used in this work. With respect to the amino acid sequence reported in the database (GenBank Accession no. K03455) [15], four substitutions (Q7K, L33I, L63I and C95A) were inserted to eliminate autoproteolysis and to avoid disulfide bridge formation. A) DsbA:HIV-1Pr fusion protein: full-length DsbA is at the N-terminal end with respect to HIV protease, whose sequence is underlined. B) GST:HIVPr-His fusion protein: GST is at the N-terminal end with respect to HIV protease, whose sequence is underlined. Bold: His-tag sequence. Bold and underlined: recognition sequence for enterokinase cleavage (A) or for PreScission protease (B).

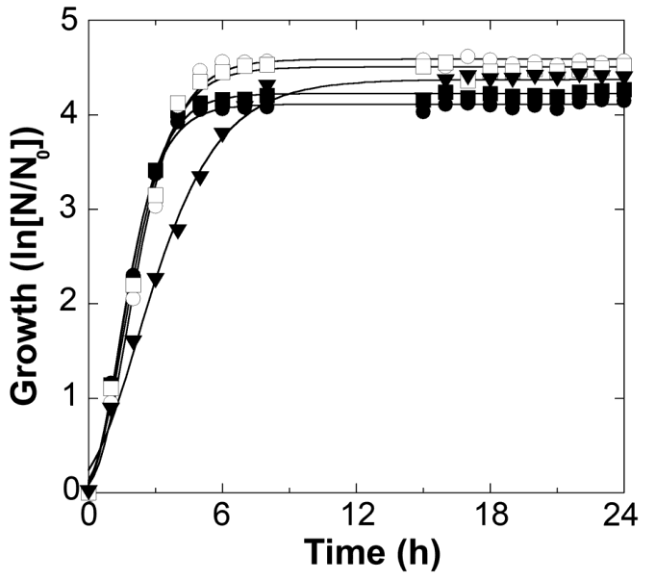

Figure S2 Growth curve of BL21-Codon Plus-(DE3)-RIL *E. coli* cells carrying the pET39-DsbA:HIV-1Pr plasmid in different media. Luria-Bertani (LB, ●), LB-Miller (■), Terrific Broth (TB, □), Super Broth (SB, ○) and M9 (▼) media. The experimental data points were analyzed by the Gompertz equation [18]. The observed specific growth rates were 1.64/h in LB, 1.47/h in LB Miller, 1.37/h in TB, 1.36/h in SB, and 0.79/h in M9. Duplication time was significantly higher in the minimal medium M9 (53 min), although the lag time is appreciably lower than in complex media.

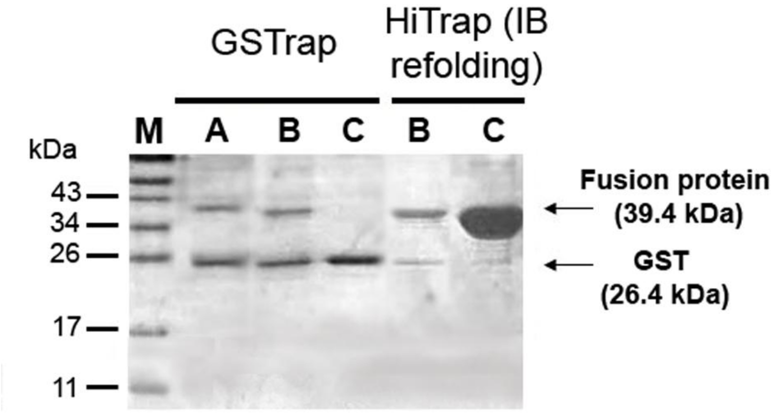

Figure S3 Purification of GST:HIV-1 Pr fusion proteins. SDS-PAGE Analysis of the purifications for the three different chimeric proteins between HIV-1Pr and GST from *E. coli* crude extract (GSTrap chromatography) or from inclusion bodies (HiTrap chelating chromatography). A) GST:HIV-1Pr; B) GST:HIVPr-His; C) His-GST:HIVPr.

Table S1 Production of recombinant HIV-1Pr in different heterologous hosts.

E. coli strain	Promoter (plasmid)	Fusion protein	Notes	Reference
BL21(DE3)	pT7 promoter (pET3amk)	β-Lactamase	- Self-processing; periplasmic	[6]
E. coli cell free			- Production: 0.43 mg/mL, soluble	Chen et al., 2007[a]
Unspecified	Unknown	-	- Additional Met at N-terminus; resolubilization from inclusion bodies by 50% acetic acid<br>- Activity: 0.27 µmol/ min mg protein	[3]
Unspecified	Various promoters	-	- Altered gene sequence (A+T richness at 5'), introduction of proteolytic autocleavage site(s) at the N-terminus; resolubilization from inclusion bodies<br>- approx. 8-10% of total cell proteins	Rangwala et al., 1992[b]
Unspecified	pL, from bacteriophage λ (PRO4 plasmid)	No fusion	- Insoluble	Stebbins and Debouck, 1994[c]
		Galactokinase	- Use of nalidixic acid as inducer<br>- Soluble, self-processing	
DH5α	ptac (pGEX-PR107)	GST	- Self-processing of chimeric protein<br>- Production: 1 mg/L<br>- Activity: < 0.4 µmol/ min mg protein	[7]
TB1	ptac (pMAL-PR107)	MBP	- Self-processing of chimeric protein<br>- Activity: < 0.3 µmol/ min mg protein	
BL21(DE3)	pT7 (pET-PR107)	No fusion	- Activity: < 0.1 µmol/ min mg protein	
JM101	placUV5 (pMON 5882, pMON 5888)	10 Amino acids of IGF-2	- Induction with 0.5 mM IPTG, growth at 42 °C for 1 hour;<br>resolubilization from inclusion bodies by 50% acetic acid<br>- Production: 20-40 mg/L pure protein	[13]

MH1	ptrp (pIFNγtrp-2)	Human γ interferon connected to HIV-1Pr by $(Asp)_4$Pro sequence	- Indole-3-acrylic acid as inducer at OD_{600} = 0.3 and growth at 37 °C for 4 hours; resolubilization from inclusion bodies	[8]
BL21(DE3)	pT7 (pT7HIVGP)	Gag-Pol presequence	- Growth at 37 °C for 3 hours or overnight after 1 mM IPTG addition at an OD_{600} = 1.0; soluble and active	[11]
DH5α	ptac (pGEX-KG)	GST	- Induction with 1 mM IPTG (and 0.5% glucose) at an OD_{600} = 0.7, followed by growth for 15' at 10 °C and for 3.5 hours at 23 °C; 60% of protein is produced as insoluble form which can be refolded<br>- Production: 1 mg/L<br>- Activity: 0.85 µmol/min mg protein	Wan and Loh, 1995[d]
JM105	pT7, placUV5 (pET3AM)	--	- Induction at an OD_{600} = 0.6, growth at 37 °C; resolubilization from inclusion bodies<br>- Activity: 0.8 µmol/min mg protein	[11]
JM109	plac (pCG8079)	MBP	- Induction at an OD_{600} = 0.5 with 1 mM IPTG, growth for 90 min at 37 °C;<br>- Production: 1 mg/L<br>- Activity: 1.1 µmol/min mg protein	Louis et al., 1991[e]
M15	Promoter inducible by IPTG (pDS 56/3H-3H)	Poly-His	- Induction at an OD_{600} = 0.7 with 0.4 mM IPTG, and then growth at 37 °C for 5 hours; resolubilization from inclusion bodies<br>-Activity: 4.4 µmol/min mg protein	[9]

IGF-2, Human insulin growth factor 2; GST, glutathione-S-transferase; MBP, maltose binding protein.

[a]Chen H, Xu Z, Yin X, Cen P: **Cloning and expression of the HIV protein in *Escherichia coli* cell free system.** *Appl Microbiol Biotechnol* 2007, **77:**347-354.

[b]Rangwala SH, Fin RF, Smith CE, Berberich SA, Salsgiver WJ, Stallings WC, Glover GI, Olins PO: **High-level production of active HIV-1**

protease in *Escherichia coli*. *Gene* 1992, **122:**263-269.

[c]Stebbins J, Debouk C: **Expression systems for retroviral proteases.** *Methods Enzymol* 1994, **241:**3-16.

[d]Wan M, Loh BN: **Expression and purification of active form of HIV-1 protease from *E. coli*.** *Biochem Mol Biol Int* 1995, **35:**899-912.

[e]Louis JM, McDonald RA, Nashed NT, Wondrak EM, Jerina DM, Oroszlan S, Mora PT: **Autoprocessing of the HIV-1 protease using purified wild-type and mutated fusion proteins expressed at high levels ion *Escherichia coli*.** *Eur J Biochem* 1991, **199:**361-369.
